# Protocol of the randomized control trial: the WiseApp trial for improving health outcomes in PLWH (WiseApp)

**DOI:** 10.1186/s12889-020-09688-0

**Published:** 2020-11-25

**Authors:** Gabriella Flynn, Haomiao Jia, Nancy R. Reynolds, David C. Mohr, Rebecca Schnall

**Affiliations:** 1 College of Public Health, University of South Florida, 13201 Bruce B. Downs Blvd, Tampa, FL 33612 USA; 2 Division of Scholarship and Research, Columbia University School of Nursing, 560 West 168th Street, New York, NY 10032 USA; 3 Johns Hopkins University School of Nursing, 525 N Wolfe Street, Baltimore, MD 21205 USA; 4 Center for Behavioral Intervention Technologies, Northwestern University, 750 N Lake Shore, Chicago, IL 60611 USA

**Keywords:** HIV/AIDS, Mobile health, Medication adherence, Randomized controlled trial, Real-time monitoring

## Abstract

**Background:**

Poor adherence to antiretroviral therapy (ART) is one of the primary barriers to viral load suppression. mHealth technology can help overcome challenges with ART adherence. This paper outlines the protocol for the WiseApp randomized control trial. The WiseApp contains real-time medication monitoring linking an electronic pill bottle and fitness tracker to the app, helping persons living with HIV (PLWH) self-manage their medication adherence and improve their overall quality of life. The primary objective of the trial is to test the effect of the WiseApp's medication adherence features on antiretroviral adherence in underserved PLWH in New York City.

**Methods:**

This ongoing study is a two-arm randomized control trial. Participants are randomized 1:1 to the WiseApp intervention arm or the control arm at baseline and followed for 6 months. Eligibility criteria include: 18 years of age, have a diagnosis of HIV, speak and understand English or Spanish, live in the United States, own a smartphone, currently taking ART medications, and report the past 30 days adherence of 80% or less as measured using the Visual Analogue Scale (VAS), or have a viral load of over 400 copies/mL. The sample size for the trial is 200 people. All study participants receive the WiseApp, a CleverCap electronic pill bottle, and a fitness tracker. The intervention group also receives videos and health surveys centered on medication adherence and managing living with HIV as well as medication reminders. In contrast, the control group receives walk step reminders, videos, and surveys focused on overall wellness.

**Discussion:**

The WiseApp Trial has the potential to improve HIV self-management applications, being one of the few randomized controlled trials of a mHealth medication adherence and HIV self-management application in the United States. The trial could also bring new opportunities for advancement in reaching economically disenfranchised and underserved populations in the United States. The real-time monitoring of the WiseApp has the potential to help providers initiate interventions to help patients resume treatment before drug resistance begins.

**Trial registration:**

This trial was registered with ClinicalTrials.gov (NCT03205982) on July 2, 2017.

## Background

An estimated 1.1 million people in the U.S. are living with HIV, with the epidemic concentrated in racial and ethnic minorities [1]. Over time, with the development of effective antiretroviral treatments, HIV has progressed to being a chronic condition with people’s life expectancy now being measured in decades [2]. However, in order for HIV to be successfully managed and maintained, persons living with HIV (PLWH) need to have access to care, begin treatment, remain in care and adhere to their antiretroviral therapy (ART) [2].

In the U.S., it is estimated that for every 100 PLWH, only 28 people successfully complete the steps to manage their care and stay in treatment with poor adherence to ART remaining the most significant challenge to treatment success [3, 4]. Insufficient engagement in care and adherence to HIV treatment leads to the progression of HIV disease and premature death among PLWH [5, 6]. New York City (NYC) accounts for 82% of all PLWH in New York State, and in the NYC region only 67% of PLWH achieve viral suppression from successful adherence to antiretroviral therapy [7]. Despite disparate populations being the most likely to be infected with HIV, these populations are less likely to adhere to their medication regimens [8]. Creating effective interventions to prevent loss of virologic control, drug resistance, and loss of treatment options as a result of non-adherence is crucial [9, 10].

mHealth technology can be useful for the management of chronic illnesses, including HIV, helping overcome the challenges of adherence to ART [11]. Further, given the ubiquity of mobile devices, mHealth has the potential to be an effective platform for the delivery of medication adherence interventions [12]. In response, we designed the WiseApp, a self-management application (app) that contains real-time medication monitoring, linking an electronic pill bottle and fitness tracker to the app, helping PLWH self-manage their medication adherence and improve their overall quality of life. The WiseApp draws on formative work with HIV Clinicians, PLWH, HIV Case Managers and the Centers for Disease Control and Prevention [13]. We refined the app for PLWH through a three-step usability evaluation using a think-aloud protocol with end-users, heuristic evaluations, and an end-user cognitive walkthrough [14]. The WiseApp is currently being tested in a randomized control trial (RCT) with PLWH in New York City and registered with ClinicalTrials.gov (NCT03205982).

### Study objective

The primary objective of this RCT was to test the effect of the WiseApp’s medication adherence features on ART adherence in underserved PLWH in NYC [14]. This paper provides an overview of the WiseApp RCT protocol.

### Ethics and consent

The Columbia University Institutional Review Board reviewed and approved all study procedures. Participants provided written consent and HIPAA authorization before enrollment, and all data collection and management procedures are collected in accordance with University policy.

## Methods/design

### Study design

This study is a two-arm RCT among racially and ethnically diverse underserved PLWH. Participants are randomized to the WiseApp intervention arm or the control arm at baseline and followed for 6 months. The differences between intervention and control arms are summarized in the description of the intervention section and outlined in Table 1.

**Table 1:** Comparison of Intervention vs. Control Group

	Intervention	Control
The Wise App		
• Medication Reminders	X	
The WiseApp		
• Step Goal Reminder		X
CleverCap^TM^ Lite dispenser	X	X

### Recruitment and eligibility

Recruitment consists primarily of community outreach, posting flyers as well as word of mouth. Community outreach is conducted in the five boroughs of NYC, visiting various community and HIV resource centers, and distributing flyers in underserved communities. Flyers also distributed at New York-Presbyterian, and the WiseApp project is listed on the Columbia University Irving Medical Center’s (CUIMC) RecruitME website, a CUIMC research database listing all actively enrolling research studies at CUIMC. Study participants also receive a study flyer to share with individuals in their community.

**Fig. 1:**
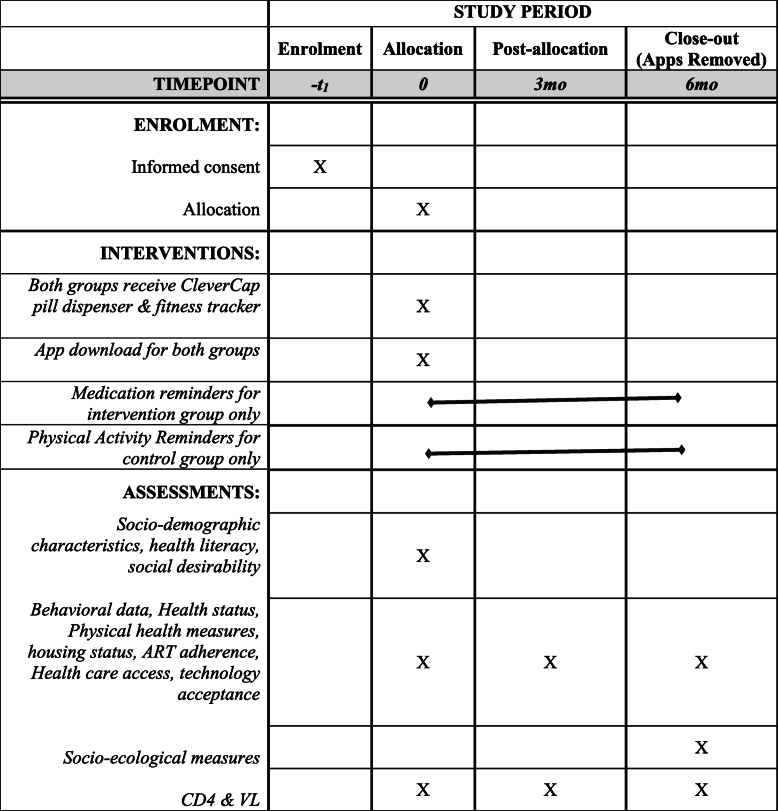
WiseApp schematic diagram

In order to be eligible, participants must be 18 years of age and older, have a diagnosis of HIV, speak and understand English or Spanish, live in the U.S., own a smartphone, and currently take ART medications. Additionally, participants must report the past 30 days adherence of 80% or less as measured using the Visual Analogue Scale (VAS) or have a viral load of over 400 copies/mL. Exclusion criteria include participation in any other mobile app study for PLWH, currently participating in a directly observed therapy program, not passing the mini-mental state neurocognitive assessment, and an inability to use apps on their phone [15]. Before participating in any study screening procedures, participants must voluntarily provide verbal informed consent. If found eligible after prescreening, participants provide written consent for the RCT during their first study visit before beginning study procedures. Our goal is to enroll 200 PLWH with 172 participants currently enrolled in the trial. We project that we will close recruitment in the Summer of 2021. (Fig. 1).

### Randomization

Study participants are randomized (1:1) to the WiseApp intervention or the control arm with a variable permuted randomized block design with the block size randomly selected between blocks of four to eight [16]. The treatment assignments in the block design were predetermined before beginning the RCT and have remained static throughout the trial. Random assignments are concealed from the participants for the duration of their participation while study staff members are aware of the participant’s randomization at baseline. At the baseline visit, study staff open a sealed study envelope and create the WiseApp profile that aligns with the treatment assignment listed inside the envelope for the baseline visit.

### Description of intervention: the WiseApp

**Fig. 2:**
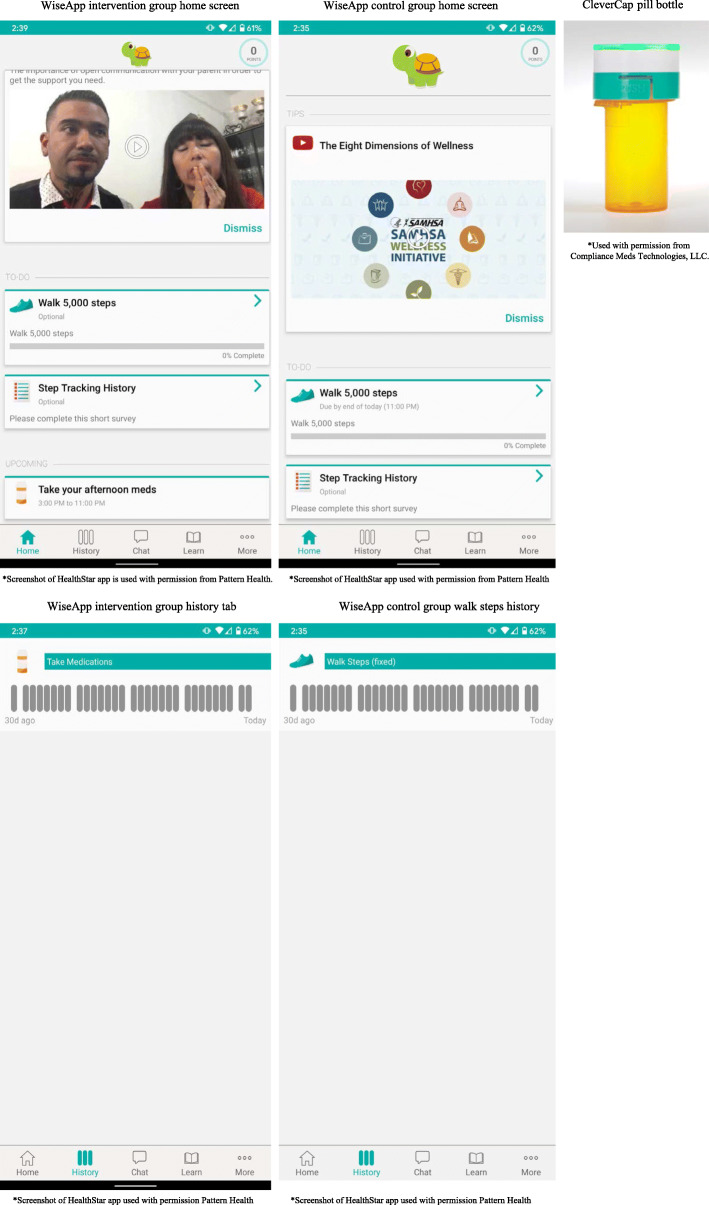
WiseApp study materials: WiseApp, CleverCap pill bottle, and fitness tracker. The intervention group receives videos and health surveys; all centered on medication adherence and managing living with HIV. A summary comparing the intervention vs. the control group is outlined below in Table 1. The intervention group’s “To Do” list serves as daily reminders for medication adherence tracking, walk steps, and completing their weekly videos and surveys. As mentioned, the CleverCap pill bottle and the fitness tracker are both linked to the WiseApp. The history tab on the WiseApp shows the intervention group’s medication adherence with green bars showing that they completed taking their medication for the day and red bars showing when they missed a day (Fig. 2). They receive daily app notification reminders for taking their medication

All study participants receive the WiseApp, a CleverCap electronic pill bottle, and a fitness tracker (Fig. 2). The WiseApp originated from formative work to design a self-management app for PLWH and was guided by Fogg’s functional triad for computing technology model [17, 18]. The WiseApp includes the following functional components: testimonials of lived experiences, push-notification reminders, medication trackers, health surveys, chat rooms, and a “To-Do” list outlining tasks for the day. Both study arms receive a fitness tracker that connects to the WiseApp. In the WiseApp, both arms also have a history tab to monitor whether or not they completed their assigned daily goals. Both study arms also receive the CleverCapp pill bottle, with only the intervention group linking the pill bottle to the WiseApp.

 The control group receives videos and health surveys centered on wellness and promoting a healthy lifestyle through diet, sleep, exercise, etc. Their “To Do” list consists of daily walk step and completing their weekly videos and surveys. Researchers can track a control member’s usage of the pill bottle through the CleverCap platform. Their history tab shows the control group’s adherence to meeting their daily step goal of 5000 steps per day. The goal of 5000 steps was selected as less than 5000 steps is often used as a sedentary lifestyle index that can be associated with cardiometabolic risks [19, 20]. The control group receives daily reminders to meet the walk step goals.

### Study assessments

  Participant complete a survey at each study visit administered through Qualtrics software. All study data are securely stored in a limited access database by study ID. All hard copy participant information (e.g., study checklists, consent forms) are securely stored at each study site in locked file cabinets with limited access. Participants are enrolled in person and complete three in-person study assessment visits over their 6 months in the RCT. At Baseline, 3 month, and 6 month follow-ups, participants have their blood drawn to measure CD4 and viral load. Participants also have the option of providing recent lab results of their CD4 and viral load data taken within the last 1–2 weeks.

In addition to completing a blood draw, participant height, weight, hip, and waist measurements are collected following standard NHANES waist and hip measurement protocol [31, 32]. Furthermore, participants complete a survey on an iPad. A full list of the study measures and tools with their measurement time points are listed in Table 2.

**Table 2:** Study measures

	Tool	Measurement time points
Descriptive Data: Socio-demographic characteristics	Gender, age, education, income, employment, health insurance, housing	Baseline
Descriptive Data: Behavioral Data	Condom Use, Tobacco Use, Alcohol Use (AUDIT-C) [21], Substance Use [22]	Baseline, 3 and 6 months
Descriptive Data: Health Literacy	Newest Vital Sign [23], Short Test of Functional Health Literacy in Adults (S-TOFHLA) [24]	Baseline
Descriptive Data: Health Status	RAND-36 [25]; Symptom Distress Module [26]; PROMIS-29 [27]; Self- Management Scale [28]; Baecke Questionnaire for the Elderly [29]	Baseline, 3 and 6 months
Descriptive Data: Social Desirability	Social desirability scale [30]	Baseline
Secondary Outcome: Physical Health Measures	Height, Weight, and NHANES waist and hip measurement protocol [31, 32].	Baseline, 3 and 6 months
Descriptive Data: Housing Status	Housing Status Assessment Tool [33]	Baseline, 3 and 6 months
Primary Outcome: ART Adherence	Medication Adherence measured through the CleverCap™ Lite Dispenser Pillbox	Daily
		Baseline, 3 and 6 months
		Baseline, 3 and 6 months
Secondary Outcomes: ART adherence	CASE Adherence Index [34]	
	CD4 and viral load	
Secondary Outcome: Health Care Access	Number of Primary Care Visits	Baseline, 3 and 6 months
	Engagement with Health care Provider Scale [35]	Baseline, 3 and 6 months
	Caregiver Survey	6 months
Secondary Outcome: System Use	Automated Log Files	Ongoing
Secondary Outcome: Technology Acceptance	Health-ITUES [36]; PSSUQ [37];	Baseline, 3 and 6 months
	Perceived Ease of Use and Potential Usefulness Questionnaire [38];	
	eHEALS: the Health Literacy Scale [39]	3 months
	Trust in Health Information Sources [40]	6 months
Secondary Outcome: Social-Ecological Measures	Neighborhood Environment Survey [41], Social Capital Scale [42], & Self- Efficacy Scale [43]	6-months

#### Outcomes

The primary outcome of the RCT is ART adherence. The CleverCap pill dispenser is the primary tool used to measure medication adherence. In addition, we use the Center for Adherence Support Evaluation (CASE) Adherence Index, and CD4 and viral load values to estimate ART adherence. The CleverCap pill dispenser automatically records each time a participant opens the pill bottle. Data collection on the opening of the pill bottle is captured each day for 6 months. To validate the findings from the CleverCap dispenser, both CD4 and VL are processed from the blood draw at each visit, and participants complete a self-report measure for adherence using the CASE Adherence Index. This adherence index consists of three questions with items scoring at a higher value indicating better adherence [44]. The secondary outcomes are: healthcare access, system use, technology acceptance, and physical activity measures. A list of all the tools to measure the secondary outcomes are listed above in Table 2.

### Statistical analysis

#### Sample size calculation

The targeted enrollment is 200 PLWH who are less than 80% adherent to their ART medications. We estimate that this will have greater than 80% power to detect at least a 10% difference in adherence to ART medication between the WiseApp intervention and the control arm*.* We made the following assumptions in our sample size and power calculation: a 75% retention rate by the end of the trial for both the control and intervention arms and that each person is on a once-daily regimen, a conservative assumption of high intraclass correlation coefficient (ICC) of 0.5 for same participant at different times, and the adherence rate is less than or equal to 80% at baseline. All calculations are based on a 2-sided test with alpha at 0.05 levels and power calculations being based on ART adherence.

Primary study data will be analyzed after completion of data collection, with study findings disseminated in peer-review public health journals.

#### Data analysis for RCT

Intention-to-treat principle will be applied for the primary outcome analysis. We purpose using a generalized linear mixed model (GLMM) to analyze primary outcome (adherence and secondary outcomes). For adherence outcome, a GLMM with logit link function will be used. In this model, independent variables are time (t) at three time points (baseline, 3 month, and 6 month), intervention arm, and interaction term between time and intervention. For the secondary outcomes in the analysis of the surveys related to the SDT framework, a similar GLMM with identity link function for continuous outcomes. In the analysis of app use, the unit of analysis will be at daily level for each participant, and system use will be analyzed using a GLMM with log link function (Poisson model).

For CD4 and viral load, we propose using GLMM with log link function because these variables are treated as count outcomes. Viral load will also be treated as a binary outcome (detectable vs. undetectable) and analyses of viral suppression will use the GLMM with logit link. In a secondary “as treated” analyses, missing adherence and viral load data will be ignored. Finally, we will assess the relationship between adherence (independent variable) and virologic suppression (dependent variable) using GLMM logistic regression and including missing viral load as detectable. This model will include personal level factors as covariates so we can test for potential confounding by covariates (i.e., age, gender, and health literacy) and inspect for changes in the point estimate of the relationship between study group and adherence. Primary study data will be analyzed as soon as possible after the end of data collection, with study findings disseminated in peer-review public health journals.

## Discussion

This protocol describes a randomized controlled efficacy trial using a mHealth intervention to help improve ART adherence and overall quality of life of PLWH living in  NYC. Previously, mHealth tools to help with HIV management, specifically adherence, have been primarily studied outside the United States in low and middle-income countries [45, 46]. A strength of our study is that, to our knowledge, this is one of the first randomized controlled trials assessing a mHealth application intervention for medication adherence and HIV management in the United States. The app was developed through user-centered design, having end-users intricately involved in the design iteration process with the only feature of the app being manipulated being the adherence features, controlling for participants receiving a health app [14]. Another strength of the WiseApp trial is the use of real-time medication monitoring with the Clever-Cap pill dispenser. This real-time monitoring can allow for easier detection of lapses in adherence and could potentially help providers initiate interventions to help resume treatment before drug resistance begins, something not previously done [47].

However, as a randomized controlled trial, there are limitations, such as detecting if differences in usage of the intervention affected outcomes. Additionally, part of this trial is occurring during the global COVID-19 pandemic. This pandemic could affect people’s usage of the app intervention due to extenuating outside stressors drawing their attention away. Some participants have also expressed difficulty in renewing medication during the pandemic.

Despite the limitations that come with an RCT, the WiseApp trial has the potential to fill a gap in the mHealth literature and provide new knowledge regarding the effectiveness of mobile app interventions for PLWH in the United States. A systematic literature review of impact studies of mHealth for HIV treatment has already found that mHealth can be effective in improving ART adherence in low and middle-income countries [46]. In another review of 721 articles, there were only six studies that reported mHealth interventions assisting with antiretroviral (ART) medication adherence among HIV-positive men who have sex with men [48]. Of the six studies, only two used mobile applications for ART and are only in the pilot phase and the beginning phases of a RCT [48]. If successful this study will not only expand the literature but also bring new advances for reaching underserved populations in the United States, improving both their adherence to HIV treatment regimens and advancement in the HIV treatment cascade.

## Supplementary information

**Additional file 1.** Wise App Trial Baseline.

## Data Availability

Not applicable.

## Abbreviations

PLWH: Persons living with HIV
HIV: Human immunodeficiency virus
ART: Antiretroviral therapy
RCT: Randomized control trial
GLMM: Generalized linear mixed model
CUIMC: Columbia University Irving Medical Center
VL: Viral load
