# Supplementary material for: Protocol of the randomized control trial: the WiseApp trial for improving health outcomes in PLWH (WiseApp)
Source: BMC Public Health. 2020 Nov 25;20:1775. doi: 10.1186/s12889-020-09688-0 (PMC7686828; doi:10.1186/s12889-020-09688-0)
Supplement: Supplementary file 1 — Additional file 1. Wise App Trial Baseline. [file 12889_2020_9688_MOESM1_ESM.pdf]

Wise App Trial Baseline

Start of Block: Introduction

*

qs_id
WiseApp Baseline
User ID

____

Page Break

End of Block: Introduction

Start of Block: HIV Meds

HIV-meds Please enter all HIV medications you are currently taking:

- ☐ Abacavir (1)
- ☐ Agenerase (2)
- ☐ Aptivus (3)
- ☐ Atripla (4)
- ☐ Biktarvy (5)
- ☐ Combivir (6)
- ☐ Complera (7)
- ☐ Crixivan (8)
- ☐ Delavirdine (9)
- ☐ Descovy (10)
- ☐ Doravirine (11)
- ☐ Edurant (12)
- ☐ Efavirenz (13)
- ☐ Emtriva (14)
- ☐ Epivir (15)
- ☐ Epzicom (16)
- ☐ Evotaz (17)
- ☐ Fortovase (Saquinavir) (18)

- [ ] Fuzeon (Enfuvirtide) (19)
- [ ] Genvoya (58)
- [ ] Hivid (ddc /zalcitabine) (20)
- [ ] Intelence (21)
- [ ] Invirase (22)
- [ ] Isentress (Raltegravir) (23)
- [ ] Juluca (59)
- [ ] Kaletra (Lopinavir) (25)
- [ ] Lexiva (Fosamprenavir) (26)
- [ ] Nevirapine (27)
- [ ] Norvir (Ritonavir) (28)
- [ ] Odefsey (29)
- [ ] Prezcobix (30)
- [ ] Prezista (Darunavir) (31)
- [ ] Rescriptor (delavirdine) (34)
- [ ] Retrovir (Zidovudine) (36)
- [ ] Reyataz (Atazanavir) (37)
- [ ] Selzentry (38)
- [ ] Stavudine (40)

☐ Stribild (41)

☐ Sustiva (Efavirenz) (42)

☐ Symtuza (43)

☐ Tivicay (44)

☐ Triumeq (46)

☐ Trizivir (47)

☐ Trogarzo (48)

☐ Truvada (49)

☐ Videx (ddI/didanosine) (50)

☐ Videx EC (51)

☐ Viracept (52)

☐ Viread (53)

☐ Viramune and Viramune XR* (54)

☐ Vitekta (55)

☐ Zerit (56)

☐ Ziagen (57)

☐ Other (60) ____

☐ Other (61) ____

☐ Other (62) ____

Start of Block: Demographics

hear How did you hear about this study?

- ☐ Online ad (please specify site or app): (1) ____
- ☐ HIV clinic (please specify clinic): (2) ____
- ☐ Community-based organization (please specify organization): (3) ____
- ☐ Friend or family member (4)
- ☐ Other (please specify): (5) ____

Page Break

age What is your age (in years)?

____

gender What is your current gender identity?

- ○ Male (1)
- ○ Female (2)
- ○ Transgender Male/Transman/FTM (3)
- ○ Transgender Female/Transwoman/MTF (4)
- ○ Genderqueer (5)
- ○ Other (please specify): (6) ____

sex What sex were you assigned at birth?

- ○ Male (1)
- ○ Female (0)

sexuality Which best describes your sexual orientation?

- ○ Homosexual/gay/lesbian (1)
- ○ Heterosexual/straight (2)
- ○ Bisexual (3)
- ○ Queer (4)
- ○ Asexual (5)
- ○ Other (please specify): (6) ____

relationship What is your primary relationship status? Are you...

- ○ Single (1)
- ○ In a relationship with a man (2)
- ○ In a relationship with a woman (3)
- ○ Legally married to a man or in a registered civil union/domestic partnership with a man (4)
- ○ Legally married to a woman or in a registered civil union/domestic partnership with a woman (5)
- ○ Divorced/separated from a man (6)
- ○ Divorced/separated from a woman (7)
- ○ Widowed (male partner) (8)
- ○ Widowed (female partner) (9)
- ○ Other (please specify): (10) ____

Page Break

race Do you consider yourself: (Check all that apply)

- ☐ African American/Black (1)
- ☐ American Indian or Alaska Native (2)
- ☐ Asian (3)
- ☐ Native Hawaiian or Other Pacific Islander (4)
- ☐ White (5)
- ☐ Other (please specify): (6) ____

---

hispanic Do you consider yourself Hispanic/Latino?

- ○ Yes (1)
- ○ No (0)

---

spanish Are you fluent in Spanish?

- ○ Yes, I speak and read Spanish (1)
- ○ Yes, I speak Spanish (2)
- ○ Yes, I read Spanish (3)
- ○ No (4)

---

education What is the highest degree or level of school you have completed?

- None (1)
- Elementary school (2)
- Some high school, no diploma (3)
- High school diploma or equivalent (e.g., GED) (4)
- Some college (5)
- Associate degree or technical degree (6)
- Bachelor/college degree (7)
- Professional or graduate degree (8)

work What is your current employment status? (Check all that apply)

- ☐ Working full-time (1)
- ☐ Working part-time (including seasonal, work-study, etc.) (2)
- ☐ Working off the books (not reported as taxable income) (3)
- ☐ Unemployed, looking for work (4)
- ☐ Unemployed, not looking for work (5)
- ☐ Retired (6)
- ☐ Student (7)
- ☐ Disabled (8)

income_annual My annual income (from all sources) is:

- Less than $10,000 (1)
- $10,000-$19,999 (2)
- $20,000-$39,999 (3)
- $40,000-$59,999 (4)
- $60,000-$79,999 (5)
- $80,000-$99,999 (6)
- $100,000-$149,999 (7)
- $150,000 or more (8)
- Don't know (9)

money_notenough In the **last 12 months**, how many times was there NOT enough money in the household for rent, food, or utilities (e.g., gas or electric bill)?

- Never (0)
- 1 time (1)
- 2 times (2)
- 3 times (3)
- 4 times (4)
- 5 times (5)
- 6 times (6)
- 7 times (7)
- 8 times (8)
- 9 times (9)
- 10 times (10)
- 11 times (11)
- 12 times (12)
- More than 12 times (13)
- I don't know (14)

moving In the **last 12 months**, how many times did you move?

- 0 (0)
- 1 (1)
- 2 (2)
- 3 or more times (3)

---

children Do you have children?

- Yes (1)
- No (2)
- Other (please describe): (3) ____

Page Break

healthinsur Do you currently have health insurance? (Check all that apply)

- ☐ Yes - Through my job (1)
- ☐ Yes - Through someone else's job (2)
- ☐ Yes - Through a health exchange (Affordable Care Act) (3)
- ☐ Yes - Paid for by me or another person (4)
- ☐ Yes - Medicaid/Medicare (5)
- ☐ Yes - ADAP (6)
- ☐ Yes - Veteran's Association (7)
- ☐ Other (please specify): (8) ____
- ☐ ☐ No (9)
- ☐ ☐ I don't know (10)

---

med_forms How confident are you filling out medical forms by yourself?

- ○ Extremely (1)
- ○ Quite a bit (2)
- ○ Somewhat (3)
- ○ A little bit (4)
- ○ Not at all (5)

diag3mo In the **last 3 months**, were you diagnosed with any of the following? (Check all that apply)

- ☐ Chancroid (1)
- ☐ Chlamydia (2)
- ☐ Gonorrhea (3)
- ☐ Hep A (4)
- ☐ Hep B (5)
- ☐ Hep C (6)
- ☐ Herpes - Genital (7)
- ☐ HPV - Genital/Anal Warts (8)
- ☐ Lymphogranuloma Venereum (9)
- ☐ Non-Gonococcal Urethritis (10)
- ☐ Syphilis (11)
- ☐ ☐ None of the above (0)

Page Break

disease Has a doctor or other medical provider **ever** told you that you had any of the following diseases or conditions? (Check all that apply)

- ☐ PCP (Pneumocystis Carinii Pneumonia) (1)
- ☐ Recurrent bacterial pneumonia (2)
- ☐ Cryptococcus (i.e., cryptococcal meningitis) (3)
- ☐ MAC (Disseminated Mycobacterium Avium Complex Disease) (4)
- ☐ Toxoplasmosis (infection of the brain) (5)
- ☐ Shingles (6)
- ☐ Recurrent herpes (HSV) or serious HSV infection (i.e., pneumonia) (7)
- ☐ CMV (cytomegalovirus) (8)
- ☐ Thrush (candidiasis) (9)
- ☐ Esophagitis (due to Candida, herpes, or CMV) (10)
- ☐ Retinitis (11)
- ☐ Tuberculosis (12)
- ☐ PML (Progressive multifocal leukoencephalopathy) (13)
- ☐ Lymphoma (14)
- ☐ KS (Kaposi's Sarcoma) (15)
- ☐ Anal cancer (16)
- ☐ Coccidioidomycosis (17)
- ☐ Histoplasmosis (18)

☐ Salmonella septicemia (recurrent) (19)

☐ Wasting syndrome from HIV (20)

☐ Encephalopathy (21)

☐ ☐ None of the above (0)

Page Break

diag Have you **ever** been diagnosed with any of the following? (Check all that apply)

- ☐ Alcohol abuse or dependence (1)
- ☐ Anxiety (2)
- ☐ Asperger's Syndrome or Autism Spectrum Disorder (3)
- ☐ Attention Deficit Disorder/ADHD (4)
- ☐ Bipolar or other mood disorder (5)
- ☐ Cancer (6)
- ☐ Chronic sinusitis (7)
- ☐ COPD (Chronic Obstructive Pulmonary Disease) or emphysema (8)
- ☐ Depression (9)
- ☐ Diabetes (10)
- ☐ Drug abuse or dependence (11)
- ☐ Eating disorder (anorexia, bulimia) (12)
- ☐ Epilepsy (13)
- ☐ Heart disease (14)
- ☐ High cholesterol (15)
- ☐ Hypertension (16)
- ☐ Insomnia (17)
- ☐ Low testosterone (18)

- [ ] MRSA (Methicillin-Resistant Staphylococcus Aureus) (19)
- [ ] Obsessive-Compulsive Disorder (20)
- [ ] Parkinson's Disease (21)
- [ ] Personality Disorder (22)
- [ ] Post-Traumatic Stress Disorder (23)
- [ ] Sexual dysfunction (24)
- [ ] Schizophrenia (25)
- [ ] Other (please specify): (26) ____
- [ ] None of the above (0)

Page Break

meds Are you **currently** taking any medications prescribed by your doctors? (Check all that apply)

- ▢ Antibiotics (e.g., Bactrim) (1)
- ▢ Anxiety medication (2)
- ▢ Bipolar medication (3)
- ▢ Blood pressure medication (4)
- ▢ Cancer medication or treatment (5)
- ▢ Cholesterol medication (6)
- ▢ Contraceptive pill (7)
- ▢ Depression medication (8)
- ▢ Diabetes medication (9)
- ▢ Erection medication (e.g., Viagra) (10)
- ▢ Heart medication (11)
- ▢ Herpes medication (12)
- ▢ Hormone replacement therapy (e.g., Premarin, Estrogel) (13)
- ▢ Schizophrenia medication (14)
- ▢ Seizure medication (15)
- ▢ Steroids (16)
- ▢ Testosterone (17)
- ▢ Other (please specify): (18) ____

☐ None of the above (0)

Page Break

healthutil_1a A healthcare provider could be a general doctor, a specialist doctor, a nurse practitioner, a physician assistant, a nurse, or anyone else you would see for health care.

People visit healthcare providers for many reasons including illness, injury, chronic health conditions, mental health treatment or therapy, substance use treatment, and other types of care. People also see their healthcare providers for regular visits such as check-ups in order to assess overall health, not usually prompted by a specific illness or complaint. People also routinely see their healthcare providers to get their medication prescriptions filled.

In the **last 30 days**, how many times have you visited a healthcare provider to get care for yourself for any reason (including visits to the hospital, ER, doctor's office, or clinic)?

____

*

Q377 Thinking of the times you have seen a healthcare provider in the **last 30 days,** how many of these visits were for an illness, sickness, or concern and NOT for a check-up or routine care?

____

Page Break

care Where do you **usually** go for HIV-related health care? (Check all that apply)

- Community-based clinic (1)
- Urgent care clinic (2)
- Emergency room (3)
- Hospital (4)
- Mobile van (5)
- Private doctor's office (6)
- School/college clinic (7)
- I don't have regular source of health care (8)
- I don't seek health care (9)
- Other (please specify): (10) ____

---

provider Is there one doctor, nurse or other medical provider whom you consider to be in charge of your overall HIV health care now?

- Yes (1)
- No (0)

---

last_appt When was the last time you had a health care appointment with the medical provider you consider to be in charge of your overall HIV health care?

- Last 3 months (1)
- 3-6 months ago (2)
- 6-9 months ago (3)
- 9-12 months ago (4)
- More than a year ago (5)
- I don't know (6)

Page Break

vload_test_6mo In the **last 6 months**, did you have a viral load test?

- Yes (1)
- No (2)
- I don't know (3)

vload_result What was the result of your most recent viral load test?

- My viral load was undetectable, OR (1)
- My viral load was detectable, OR >200 copies/ml (2)
- I don't know -- but I think I was detectable (3)
- I don't know -- but I think I was undetectable (4)

cd4_test_6mo In the **last 6 months**, did you have a CD4 or T-cell test?

- Yes (1)
- No (2)
- I don't know (3)

cd4 When was your **most recent** CD4 or T-cell count? Please provide the month and year.

- ○ Month (mm) (1) ____
- ○ Year (yyyy) (2) ____

cd4_result What was your **most recent** CD4 or T-cell count?

- ○ Less than 200 (1)
- ○ 201 - 349 (2)
- ○ 350 - 499 (3)
- ○ 500 or higher (4)
- ○ I don't know (5)

cd4_lowestresult What was your **lowest** CD4 or T-cell count **ever**?

- ○ Less than 200 (1)
- ○ 201 - 349 (2)
- ○ 350 - 499 (3)
- ○ 500 or higher (4)
- ○ I don't know (5)

cd4_lowest When did your **lowest** CD4 or T-cell count occur? Please provide the month and year.

- ○ Month (mm) (1) ____
- ○ Year (yyyy) (2) ____

Page Break

hivdx When were you diagnosed with HIV? Please provide the month and year.

- Month (mm) (1) ____
- Year (yyyy) (2) ____

aids Have you **ever** been diagnosed with AIDS?

- Yes (1)
- No (2)
- I don't know (3)

art Are you **currently** taking antiretroviral medications to treat your HIV infection?

- Yes (1)
- No (0)

pill Do you take one pill a day to treat your HIV infection?

- Yes (1)
- No, I take 2 or more pills a day (2)
- Not sure (3)

pillbox Where do you keep your HIV medication?

- ○ Original pill bottle (1)
- ○ Pill box (2)
- ○ Other (specify): (3) ____

Page Break

support_gettogether In the **last 3 month**s, when you needed it, how often did your friends, family or partner get together with you to relax and have fun?

- Very often (1)
- Somewhat often (2)
- Not very often (3)
- Not at all (4)
- I don't know (5)
- Prefer not to answer (6)

support_talk In the **last 3 months**, when you needed it, how often did your friends, family or partner talk with you about things that were very personal or private?

- Very often (1)
- Somewhat often (2)
- Not very often (3)
- Not at all (4)
- I don't know (5)
- Prefer not to answer (6)

religion How important is religion in your life?

- Very (1)
- Somewhat (2)
- Not very (3)
- Not at all (4)
- I don't know (5)
- Prefer not to answer (6)

Page Break

stigma_instructio Please answer whether you agree or disagree with the following statements.

---

stigma1 I have been hurt by how people reacted to learning I have HIV.

- ◯ Strongly disagree (1)
- ◯ Disagree (2)
- ◯ Agree (3)
- ◯ Strongly agree (4)
- ◯ Prefer not to answer (5)

---

stigma2 I have stopped socializing with some people because of their reactions to my having HIV.

- ◯ Strongly disagree (1)
- ◯ Disagree (2)
- ◯ Agree (3)
- ◯ Strongly agree (4)
- ◯ Prefer not to answer (5)

---

stigma3 I have lost friends by telling them I have HIV.

- Strongly disagree (1)
- Disagree (2)
- Agree (3)
- Strongly agree (4)
- Prefer not to answer (5)

stigma4 I am very careful who I tell that I have HIV.

- Strongly disagree (1)
- Disagree (2)
- Agree (3)
- Strongly agree (4)
- Prefer not to answer (5)

stigma5 I worry that people who know I have HIV will tell others.

- Strongly disagree (4)
- Disagree (2)
- Agree (3)
- Strongly agree (4)
- Prefer not to answer (5)

stigma6 Most people with HIV are rejected when others find out.

- Strongly disagree (1)
- Disagree (2)
- Agree (3)
- Strongly agree (4)
- Prefer not to answer (5)

Page Break

*

trap1 Please select the number 4 below. This helps prevent automated programs from abusing the study.

- 6 (1)
- 5 (2)
- 4 (3)
- 3 (4)
- 2 (5)
- 1 (6)

Page Break

Start of Block: Non-CTP Medical and Other Services

nms1 Are you **currently** receiving substance abuse treatment services?

- Yes (1)
- No (2)
- Prefer not to answer (3)

---

nms2
In the **past 30 days**, have you participated in an outpatient treatment program for drug or alcohol problems?

- Yes (1)
- No (2)
- Prefer not to answer (3)

Skip To: nms3 If In the past 30 days, have you participated in an outpatient treatment program for drug or alcohol... != Yes

---

Page Break

nonctp_instructions1 These questions refer to your participation in outpatient treatment program(s) for drug or alcohol problems within the **last 30 days**.

*

nms2a How many days have you participated?

____

*

nms2b How many hours do you attend the program in a typical week?

____

nms2c

Are you, or have you been, required by the criminal justice system to attend treatment?

- Yes (1)
- No (2)
- Prefer not to answer (3)

Page Break

nms3
In the **past 30 days**, have you been admitted into a residential program for detox or for other services?

- Yes (1)
- No (2)
- Prefer not to answer (3)

Skip To: nms4 If In the past 30 days, have you been admitted into a residential program for detox or for other ser... != Yes

Page Break

nonctp_instructions2 These questions refer to your admission(s) the residential programs for detox or for other services within the **last 30 days**.

*

nms3a
How many admissions?

____

*

nms3b
How many nights altogether for all stays?

____

Page Break

nms4
In the **past 30 days**, have you been admitted into a hospital for detox?

- ○ Yes (1)
- ○ No (2)
- ○ Prefer not to answer (3)

Skip To: nms5 If In the past 30 days, have you been admitted into a hospital for detox? != Yes

Page Break

nonctp_instructions3 These questions refer to your admission(s) into a hospital for detox within the **last 30 days**.

*

nms4a
How many admissions?

____

*

nms4b
How many nights altogether for all stays?

____

Page Break

nms5

In the **past 30 days**, have you been admitted to the hospital for any other reason?

- ○ Yes (1)
- ○ No (2)
- ○ Prefer not to answer (3)

Skip To: nms6 If In the past 30 days, have you been admitted to the hospital for any other reason? != Yes

Page Break

nonctp_instructions4 These questions refer to your admission(s) into a hospital for any reason other than detox within the **last 30 days**.

---

*

nms5a
How many times were you admitted?

____

---

*

nms5b
How many nights altogether for all stays?

____

---

nms5c
Were any admissions for psychiatric or emotional reasons?

◯ Yes (1)

◯ No (2)

◯ Prefer not to answer (3)

Page Break

nms6
In the **past 30 days**, have you visited an emergency room and not been admitted to the hospital?

- Yes (1)
- No (2)
- Prefer not to answer (3)

Skip To: nms7 If In the past 30 days, have you visited an emergency room and not been admitted to the hospital? != Yes

Page Break

nonctp_instructions5 This question refers to your visit(s) to an emergency room without admission to the hospital within the **last 30 days**.

nms6a How many times did you visit the emergency room without being admitted to the hospital?

____

Page Break

nms7

Outside of the services or programs mentioned above, have you seen a therapist, that is a psychiatrist, psychologist, counselor, or social worker for psychological or emotional problems in the **last 30 days**?

- ○ Yes (1)
- ○ No (2)
- ○ Prefer not to answer (3)

Skip To: nms8 If Outside of the services or programs mentioned above, have you seen a therapist, that is a psychia... != Yes

Page Break

nonctp_instructions6 This question refers to your visit(s) with a therapist for psychological or emotional problems within the **last 30 days**.

*

nms7a
How many times did you see a psychiatrist, psychologist, counselor or social worker?

____

Page Break

nms8
Outside of the services or programs mentioned above, have you seen a therapist, that is a psychiatrist, psychologist, counselor, or social worker for alcohol or drug problems in the **last 30 days**?

- Yes (1)
- No (2)
- Prefer not to answer (3)

Skip To: nms9 If Outside of the services or programs mentioned above, have you seen a therapist, that is a psychia... != Yes

Page Break

nonctp_instructions7 This question refers to your visit(s) with a therapist for alcohol or drug problems within the **last 30 days**.

*

nms8a
How many times did you see a psychiatrist, psychologist, counselor or social worker?

____

Page Break

nms9
In the **last 30 days**, have you visited a medical office, not including your therapist? (Please include all visits to a physician, nurse, nurse practitioner, or physician's assistant.)

- ○ Yes (1)
- ○ No (2)
- ○ Prefer not to answer (3)

Skip To: nms10 If In the last 30 days, have you visited a medical office, not including your therapist? (Please inc... != Yes

Page Break

nonctp_instructions8 These questions refer to your visit(s) to a medical office within the **last 30 days**.

*

nms9a
How many visits to a medical office have you had?

____

*

nms9b
How many of these visits did you see a doctor?

____

Page Break

nms10
In the **last 30 days**, have you attended Alcoholics Anonymous (AA), Narcotics Anonymous (NA), or Cocaine Anonymous (CA) meetings?

- Yes (1)
- No (2)
- Prefer not to answer (3)

Skip To: nms11 If In the last 30 days, have you attended Alcoholics Anonymous (AA), Narcotics Anonymous (NA), or Co... != Yes

Page Break

nonctp_instructions9 This question refers to your attendance at AA, NA, or CA within the **last 30 days**.

*

nms10a
For how many days?

____

Page Break

nms11
Are you **currently** prescribed any medication for the treatment of substance abuse?

- ○ Yes (1)
- ○ No (2)
- ○ Prefer not to answer (3)

Skip To: nms12 If Are you currently prescribed any medication for the treatment of substance abuse? != Yes

Page Break

nonctp_instruction10 These questions refer to your **current** prescription(s) for medication for the treatment of substance abuse.

X→

nms11a Which of the following medications have you been prescribed?

- ☐ Suboxone (1)
- ☐ Subutex/buprenorphine (2)
- ☐ Methadone (3)
- ☐ Naltrexone (4)
- ☐ Depot Naltrexone (5)
- ☐ Acamprosate (6)
- ☐ Antabuse/disulfiram (7)
- ☐ Other (please specify): (8) ____

---

*

nms11b In the **last 30 days**, how many total days did you take this/these medication(s)?

____

Page Break

nms12
In the **past 3 months**, have you spent time uninsured?

- Yes (1)
- No (2)
- Prefer not to answer (3)

---

nms13 In the **last 30 days**, have you had a job? (This includes any job for which you have been paid, including under-the-table work.)

- Yes (1)
- No (2)
- Prefer not to answer (3)

Skip To: End of Block If In the last 30 days, have you had a job? (This includes any job for which you have been paid, inc... != Yes

---

Page Break

nonctp_instruction11 These questions refer to the job(s) for which you have been paid, including under-the-table work, in the **past 30 days**.

---

*

nms13a
How many days have you been paid for working?

____

---

*

nms13b
Including overtime, how many hours per week do you work on this job?

____

---

*

nms13c
Including tips and bonuses, what is your hourly rate on this job, before taxes?

○ $ (1) ____

Skip To: End of Block If Condition: $ Is Empty. Skip To: End of Block.

Skip To: End of Block If Condition: $ Is Not Empty. Skip To: End of Block.

Page Break

nms16 Have you **ever** been incarcerated (spent the night in a jail, prison, or detention facility)?

- ◯ Yes (1)
- ◯ No (2)
- ◯ Prefer not to answer (3)

---

nms16a Have you been incarcerated (spent the night in a jail, prison, or detention facility) in the **last 3 months**?

- ◯ Yes (1)
- ◯ No (2)
- ◯ Prefer not to answer (3)

Page Break

Start of Block: COVID-19 - v1

covid_intro On February 11, 2020, an official name was announced for the disease that is causing the 2019 novel coronavirus outbreak. The new name of this disease is coronavirus disease 2019, abbreviated and commonly referred to as "COVID-19." To better understand the impacts of COVID-19, the next set of questions will ask about your perceptions of how your health has been impacted by the COVID-19 outbreak.

Remember that your answers are private and that you will never be individually identified in any report or publishing of our findings. Please answer as best you can and be as honest as possible.

Page Break

X→

covid_dx Have you been diagnosed with COVID-19 by a doctor or another health care provider?

- ○ Yes (1)
- ○ No (2)
- ○ I don't know (999)

Display This Question:

If covid_dx = 1

covid_hosp Were you hospitalized due to your COVID-19 diagnosis?

- ○ Yes (1)
- ○ No (2)

Page Break

covid_dx_others Have any of your close friends or family members been diagnosed with COVID-19 by a doctor or another health care provider?

- Yes (1)
- No (2)
- I don't know (999)

Page Break

covid_concern Overall, how concerned are you about the COVID-19 pandemic?

- Not at all concerned (0)
- A little concerned (1)
- Somewhat concerned (2)
- Very concerned (3)

Page Break

covid_order Do you currently reside in a city, county, state, or other locale that currently has or previously had a **Shelter in Place/Stay at Home Order** due to COVID-19?

- ◯ Yes (1)
- ◯ No (2)
- ◯ I don't know (999)

Display This Question:

If covid_order = 1

covid_order_follow Which government-level **Shelter in Place/Stay at Home Orders** are you following? *(Please select all that apply.)*

- ▢ City (please specify) (1) ____
- ▢ County (please specify) (2) ____
- ▢ State (please specify) (3) ____

Display This Question:

If covid_order = 2

Or covid_order = 999

vol_quarantine Even without a city, county, state, or other local Shelter in Place/Stay at Home Order, have you **voluntarily decided** to shelter in place/stay at home/self-quarantine anyway due to COVID-19?

- ◯ Yes (1)
- ◯ No (2)

Skip To: hiv_sti_test If vol_quarantine = 2

Page Break

Display This Question:

If covid_order = 2

Or covid_order = 999

vol_quarantive_actv Are you still **voluntarily** sheltering in place/staying at home/self-quarantining due to COVID-19?

○ Yes (1)

○ No (2)

Page Break

Display This Question:

If covid_order = 2

Or covid_order = 999

vol_quaratine_dates On approximately what date did your **voluntary self-quarantine** start (and end, if applicable) even though there wasn't a formal Shelter in Place/Stay at Home Order in your city, county, state, or other locale? *(Example: 05/14/2020)*

○ Start Date (MM/DD/YYYY) (1) ____

vol_quarantive_actv = 2

○ End Date (MM/DD/YYYY) (2) ____

Display This Question:

If covid_order = 1

covid_order_actv Are you still sheltering in place/staying at home/quarantining because of the Shelter in Place/Stay at Home Order imposed by your city, county, or state?

○ Yes (1)

○ No (2)

Display This Question:

If covid_order = 1

covid_order_dates On approximately what date did the Shelter in Place/Stay at Home Order start (and end, if applicable) in your city, county, state, or other locale? (Example: 05/14/2020)

○ Start Date (MM/DD/YYYY) (1) ____

covid_order_actv = 2

○ End Date (MM/DD/YYYY) (2) ____

Page Break

Page Break

covid_medcare_concer How concerned are you about your ability to access medical care during the COVID-19 outbreak?

○ Not at all concerned (0)

○ A little concerned (1)

○ Somewhat concerned (2)

○ Very concerned (3)

Page Break

ART_prior_covid Prior to COVID-19 outbreak, were you using ART?

○ Yes (1)

○ No (0)

Skip To: covid_prep_concern If prep_prior_covid = 0

Page Break

covid_ART_change Have you changed how you take ART since the COVID-19 outbreak started?

○ No, my ART use hasn't changed (0)

○ Yes, I stopped taking ART (1)

○ Yes, my ART use changed in some ways but did not stop completely (please specify) (2) ____

Skip To: prep_provider_contac If covid_prep_change = 0

Page Break

covid_stop_ART Why did you stop taking ART as often or completely?

○ I don't think I need it anymore (please specify) (1) ____

○ I don't have access to it anymore (2)

○ I stopped for some other reason (please specify) (3) ____

Skip To: covid_partners_chang If covid_stop_prep = 1

Page Break

Display This Question:

If covid_stop_prep = 2

covid_ART_noaccess Why do you not have access to ART anymore?

- I don’t have health insurance anymore (1)
- My provider can’t prescribe or refill my prescription (2)
- I can’t complete routine testing/labs required for my prescription (3)
- Some other reason (please specify) (4) ____

Page Break

X→

ART_provider_contac Have you reached out to your provider for an ART refill?

- Yes, I have (1)
- No, I have not (0)
- No, I have more than a 90-day supply (999)

Page Break

Display This Question:

If prep_provider_contac = 1

X→

covid_provider_offer Did your provider offer any of the following services when you reached out about a ART refill?

◯ My provider offered a refill without the quarterly visit (1)

◯ My provider offered a telemedicine (e.g., video call) appointment to refill my prescription (2)

◯ My provider offered an office-based appointment to refill my prescription (3)

◯ My provider offered something else (please specify (4)
____

Page Break

covid_ART_concern How concerned are you about accessing ART since the COVID-19 outbreak started?

◯ Not at all concerned (0)

◯ A little concerned (1)

◯ Somewhat concerned (2)

◯ Very concerned (3)

Page Break

End of Block: COVID-19 - v1
